# Sphingosine‐1‐Phosphate Promotes FOS Activation in Osteosarcoma Under Tumor Acidosis

**DOI:** 10.1111/apha.70214

**Published:** 2026-04-26

**Authors:** Nicolò Bozzini, Margherita Cortini, Alberto Righi, Agamemnon E. Grigoriadis, Michael Dack, Elizabeta Ilieva, Federica Torricelli, Veronica Manicardi, Nicola Baldini, Sofia Avnet

**Affiliations:** ^1^ Department of Biomedical and Neuromotor Sciences University of Bologna Bologna Italy; ^2^ Department of Pathology IRCCS Istituto Ortopedico Rizzoli Bologna Italy; ^3^ Centre for Craniofacial and Regenerative Biology King's College London, Guy's Hospital London UK; ^4^ Laboratory of Translational Research Azienda USL‐IRCCS di Reggio Emilia Reggio Emilia Italy; ^5^ Biomedical Science, Technologies, and Nanobiotechnology Lab IRCCS Istituto Ortopedico Rizzoli Bologna Italy

**Keywords:** acidosis, epigenetics, FOS, osteosarcoma, sphingosine kinase 2, sphingosine‐1‐phosphate, tumor microenvironment

## Abstract

**Aim:**

The tumor microenvironment in solid tumors is characterized by extracellular acidosis, which promotes cancer aggressiveness. In osteosarcoma, the most common primary bone cancer, a highly acidic tumor microenvironment is associated with metastasis and poor prognosis, partly due to metabolic rewiring, including changes in lipid pathways such as those involving sphingosine‐1‐phosphate, a bioactive sphingolipid. Sphingosine‐1‐phosphate has been previously implicated in histone deacetylase inhibition and gene activation. Here, we investigated whether acidosis induces nuclear sphingosine‐1‐phosphate accumulation via sphingosine kinase 2, leading to epigenetic activation of oncogenes like *FOS* in osteosarcoma.

**Methods:**

Osteosarcoma spheroids were cultured under neutral or acidic conditions. Histone H3 acetylation was assessed by capillary Western blotting. *FOS* expression and FOS nuclear localization were analyzed. Sphingosine‐1‐phosphate's role was addressed through sphingosine kinase 2 silencing and inhibition (ABC294640). Functional effects were measured using colony formation assays. Patient‐derived OS tissues (*n* = 7) were analyzed for correlations between acidity markers (LAMP2, V‐ATPase), sphingosine kinase 2, and *FOS* expression.

**Results:**

Acidosis increased both sphingosine kinase 2 mRNA expression after 24 h and histone H3 acetylation, which followed progressive *FOS* upregulation and nuclear FOS accumulation. Sphingosine kinase 2 inhibition or silencing reduced these effects and impaired clonogenicity. In patient tissues, sphingosine kinase 2 levels correlated with acidosis markers and FOS expression.

**Conclusions:**

We identified a novel mechanism where acidosis stimulates both nuclear sphingosine kinase 2 to synthesize sphingosine‐1‐phosphate and histone H3 acetylation, ultimately leading to *FOS* transcription. Targeting this axis decreased clonogenesis, underscoring its therapeutic potential in osteosarcoma and potentially other acid‐adapted cancers.

**Abbreviations**
ECM: extracellular matrix
HDAC: histone deacetylase
hOB: normal osteoblasts
LD: lipid droplets
OS: osteosarcoma
S1P: sphingosine‐1‐phosphate
SEM: standard error of the mean
SphK: sphingosine kinase
TGF‐β2: transforming Growth Factor beta2
TME: tumor microenvironment

## 1 Introduction

Tumors are complex ecosystems composed of cancer cells, stromal cells, secreted factors, and the extracellular matrix (ECM), collectively referred to as the tumor microenvironment (TME). The TME plays a critical role in tumor survival, progression, and therapeutic resistance, making it a central focus in cancer research [1, 2, 3, 4]. A hallmark of the TME is extracellular acidosis, frequently observed in advanced solid tumors [5]. This condition arises from metabolic reprogramming, including increased glycolysis in poorly perfused or highly proliferating cells, elevated activity of H^+^‐ATPases and Na^+^/H^+^ exchangers, and CO_2_ production by oxidative metabolism [6, 7, 8]. Acidosis has a profound impact on cancer cell metabolism, gene expression, and epigenetic regulation. It contributes to tumor aggressiveness, treatment resistance, and cellular quiescence [4, 9, 10, 11]. For example, acidosis has been shown to induce deacetylation mediated by sirtuins 1 and 6, thus leading to reduced expression of acetyl‐CoA carboxylase 2. This inhibits fatty acid degradation while allowing simultaneous fatty acid synthesis and oxidation [6, 12]. One major consequence of acidosis is the rewiring of lipid metabolism [13, 14, 15]. While lipid droplet (LD) formation is typically triggered by nutrients and redox imbalances, acidosis also stimulates fatty acid uptake and triglycerides accumulation in LD via transforming growth factor beta 2 (TGF‐β2) signaling [14], and has more recently been shown to directly enhance transporter‐independent fatty acid uptake under acidic conditions, supporting a complementary physicochemical mechanism for acidosis‐driven lipid storage [12, 16, 17, 18]. This adaptation may serve as both an energy reservoir and a defense mechanism against lipotoxicity [19].

Osteosarcoma (OS) is the most common primary malignant bone tumor in children and adolescents [15]. It is highly aggressive and often leads to lung metastases, despite intensive multi‐agent chemotherapy. As a result, OS remains associated with a poor outcome [20]. Like many other solid tumors, OS creates an acidic TME, which promotes metastasis and treatment resistance [21, 22, 23], with a mechanism that is not completely understood. In a recent lipidomic analysis, we identified an acid‐induced upregulation of sphingomyelins and sphingosine‐1‐phosphate (S1P) in OS [24]. Sphingolipids, particularly ceramide and S1P, function as bioactive signaling molecules in several cancers [24]. S1P supports cell survival, proliferation, and migration through G‐protein coupled S1P receptors [25]. It is synthesized by sphingosine kinase‐SphK1 in the cytoplasm and SphK2 in the nucleus [15]. We previously showed that S1P is elevated in the serum of patients and its pharmacological inhibition enhances OS cell survival and migration in vitro [15]. Importantly, nuclear S1P, produced by SphK2, can also regulate the epigenome [26]. In MCF‐7 and HeLa cells, nuclear S1P inhibits histone deacetylases 1 and 2 (HDAC1/2), leading to increased acetylation of histone H3K9, H4K5, and H2BK12 [27, 28]. This epigenetic shift activates the transcription of key genes, including the oncogene *FOS*, the cyclin‐dependent kinase inhibitor p21, and hypoxia‐inducible factor 1α. In the same study, SphK2 has also been found within repressive chromatin complexes at these loci, suggesting that locally synthesized S1P may selectively inhibit nearby HDACs [27]. Based on these findings, we hypothesize that extracellular acidosis in the TME induces lipid accumulation, particularly S1P. In turn, S1P may act as an epigenetic regulator in response to microenvironmental stress, activating selective oncogenes that drive tumor progression. In OS, key oncogenes include *TP53, RB1, MYC, MDM2*, and *FOS* [29, 30]. Among these, *FOS* is highly expressed in both primary tumors and lung metastases [31]. Elevated FOS levels disrupt normal osteoblastic cell growth and promote a tumorigenic phenotype [32] and prior studies have also highlighted that S1P can regulate FOS expression through epigenetic mechanisms [27].

In this study, we investigated whether acidosis‐induced S1P accumulation drives the epigenetic activation of oncogenes in OS, with a focus on FOS. We validated our findings in vitro and in patient‐derived metastatic OS samples. We also assessed the potential of targeting this pathway as a therapeutic strategy.

## 2 Results

### 2.1 Extracellular Acidosis Induces H3 Acetylation via S1P in 143B OS Cells In Vitro

We sought to investigate whether tumor‐derived extracellular acidosis could act as an autoregulatory mechanism for cancer progression through the induction of histone acetylation and oncogene transcription in OS. As in vitro models, we used 3D tumor spheroids derived from two representative OS cell lines: the highly metastatic 143B cells and the less aggressive MG‐63. The acidic tumor microenvironment was simulated using buffered neutral pH 7.4, buffered acidic (pH 6.8), or unbuffered culture conditions. Direct pH measurements confirmed the stability of the buffered conditions throughout culture, whereas the unbuffered system exhibited a gradual decrease from approximately pH 6.8 to 6.5 over 96 h, reflecting metabolically driven acidification consistent with physiological intratumoral pH ranges (Figure S1) [33, 34].

**FIGURE 1:**
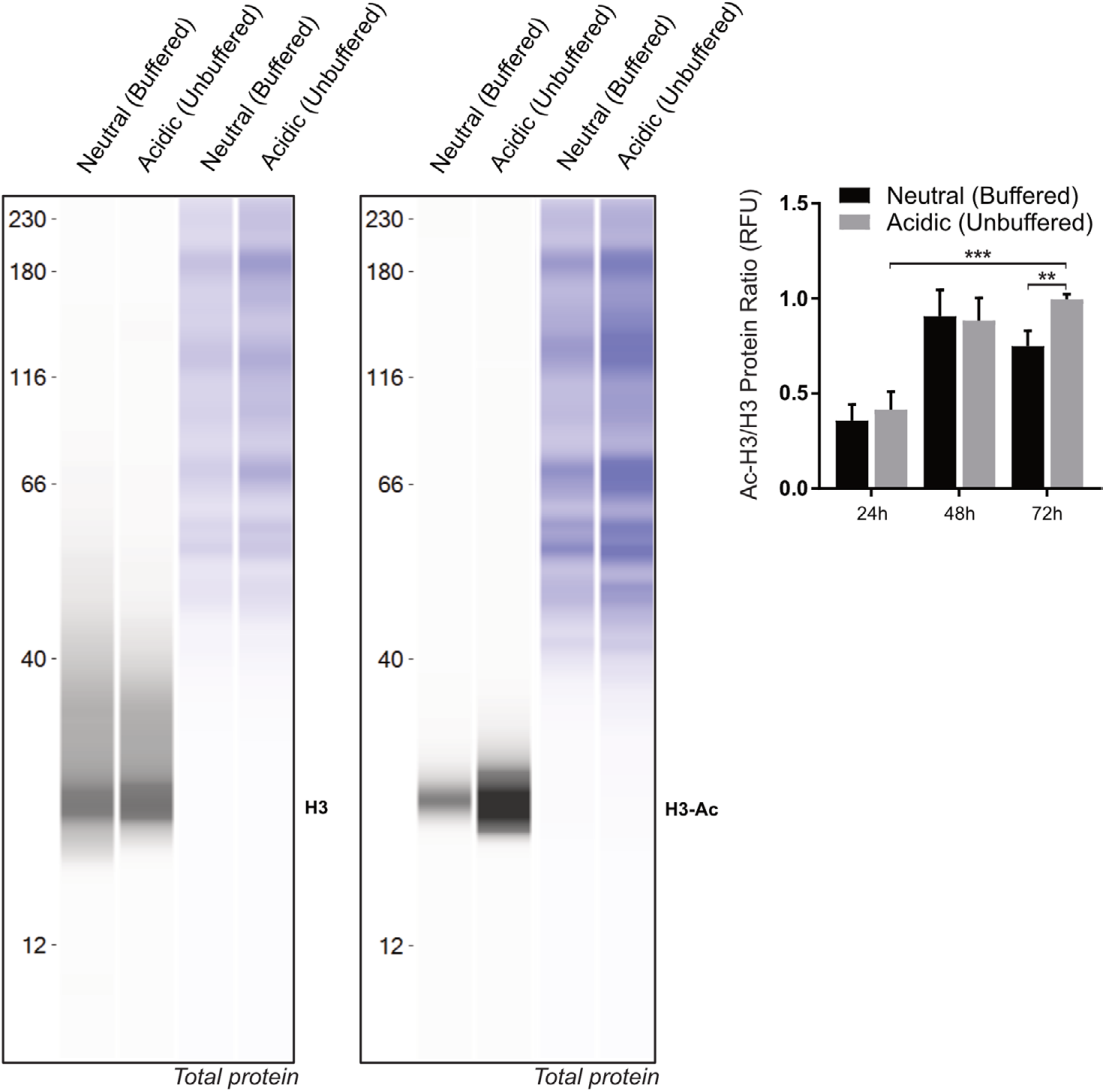
Acidosis increases H3 acetylation in 143B spheroids. Left panel, representative capillary western blot showing the signals of H3 and H3‐Ac in 3D spheroids under neutral vs. acidic (unbuffered) conditions, at 72 h (total protein in the respective right panel); right panel, acetylated/non‐acetylated H3 ratio quantification at 24 h–48 h–72 h (**p < 0.01, ***p < 0.001, *n* = 6). One‐tailed Mann–Whitney test, mean ± SEM.

We performed capillary Western blotting to assess total H3 and acetylated H3 (H3‐Ac) levels in cell cultured under neutral or acidic conditions at different time points. In 143B cells, we observed a trend of progressive increase in H3 acetylation over time, reaching the highest average value at 72 h. At this time point, H3 acetylation was significantly higher under acidic conditions compared with neutral pH (Figure 1, Figure S2A, *p* = 0.0008 and *p* = 0.0015, respectively). Importantly, a similar increase in H3 acetylation was also observed in cells cultured under buffered pH 6.8 conditions (Figure S3), confirming that the effect is reproducible in controlled acidic environments. All signals were within the linear range of the assay, and H3‐Ac/H3 ratios were calculated from normalized peak areas rather than visual band intensity to ensure accurate quantification. In contrast, MG‐63 cells showed stable H3‐Ac/H3 ratios, with no significant differences between neutral and acidic conditions (Figure S4).

These results suggest that extracellular acidosis promotes histone H3 acetylation.

### 2.2 Extracellular Acidosis Promotes FOS Expression via SphK2‐Dependent S1P Signaling in Vitro

**FIGURE 2:**
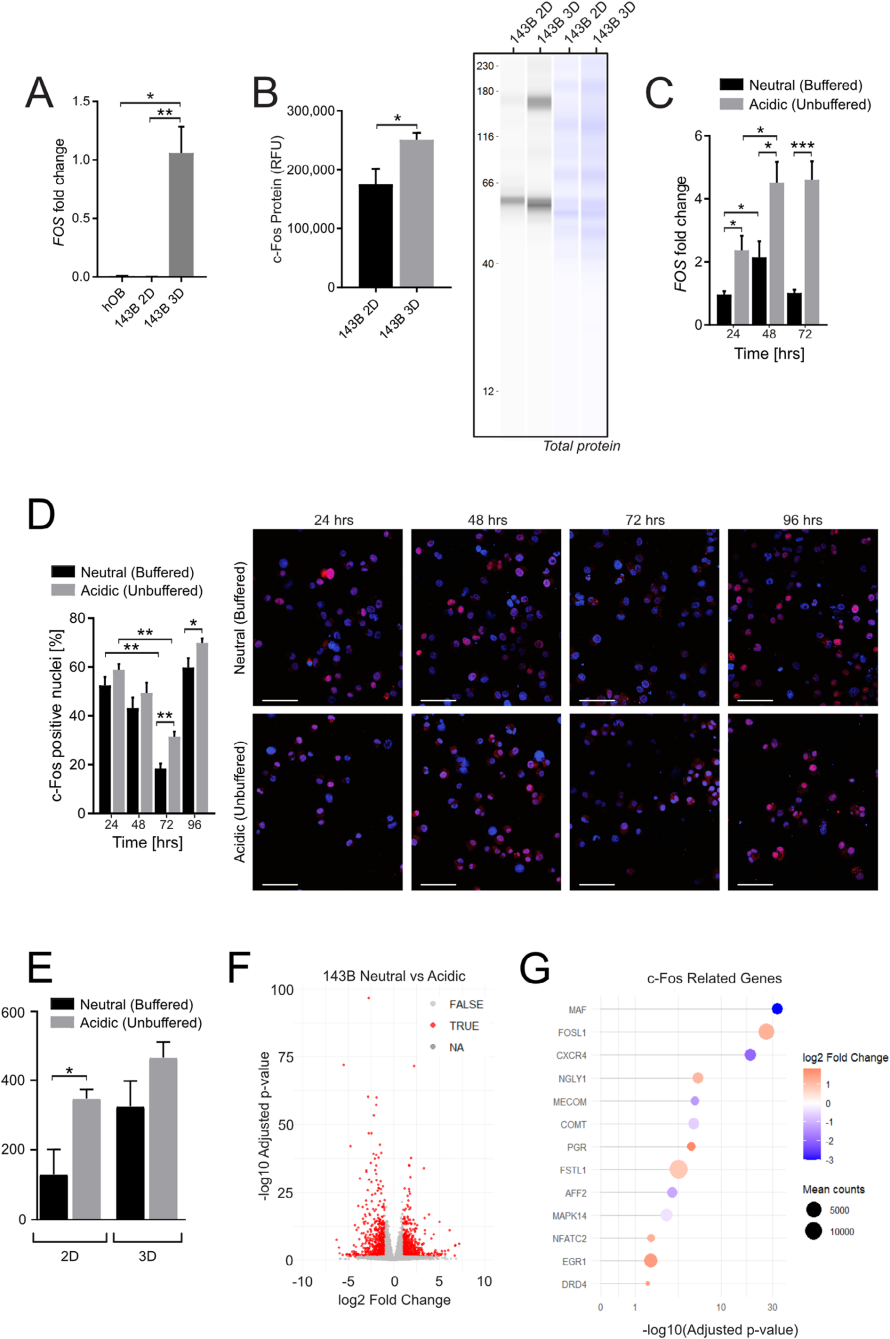
Acidosis promotes S1P‐mediated upregulation of FOS expression and nuclear localization, in vitro. (A) Q‐RT‐PCR of *FOS* mRNA in healthy osteoblasts (hOB), 2D 143B cells, and 3D 143B spheroids, normalized to GAPDH (*p < 0.05, **p < 0.01, *n* = 4); (B) Simple Western blot quantification of FOS protein in 2D and 3D 143B cells, normalized to total protein (RFU) (*p < 0.05, *n* = 4); (C) Q‐RT‐PCR of *FOS* mRNA in 143B spheroids in neutral vs. unbuffered medium over time, normalized to GAPDH (*p < 0.05, ***p < 0.001, *n* = 4); (D) Left: Representative FOS immunofluorescence in 143B spheroids in neutral vs. acidic (unbuffered) medium over time by immunofluorescence (red, nuclei were counterstained with bisBenzimide H 33258) (scale bar 100 μm); Right: FOS positive nuclei (%) (*p < 0.05, **p < 0.01, ***p < 0.001, *n* = 5); (E) Quantification of FOS transcription from transcriptomic analysis in neutral vs. acidic (unbuffered) conditions (*p < 0.05, *n* = 6); (F) Volcano plot of differential gene expression between 143B cells cultured in neutral and acidic (unbuffered) conditions from transcriptomic analysis; (G) Lollipop plot of differentially expressed genes related to FOS from transcriptomic analysis.

We next investigated whether extracellular acidosis modulates *FOS* expression in OS. We examined *FOS* mRNA expression and FOS protein levels in 143B OS cells, cultured in both 2D and 3D conditions, and compared them to normal osteoblasts (hOB). *FOS* mRNA expression was markedly elevated in OS cell spheroids compared with normal osteoblasts in which it was almost absent, and the 3D spheroids also showed higher expression versus 2D cell cultures (Figure 2A, *p* = 0.0079 and *p* = 0.0143, respectively). At protein level, this significant difference was maintained (Figure 2B, Figure S2B, *p* = 0.00286). These findings suggest that 3D cultures better mimic the in vivo tumor environment and validate their use for further mechanistic study.

We then assessed whether extracellular pH affects *FOS* expression over time. In 143B spheroids, acidic conditions significantly stimulated *FOS* expression at all the time points observed (Figure 2C, *p* = 0.0143), indicating that acidosis stimulates FOS transcription. Given that FOS functions as a nuclear transcription factor and translocates to the nucleus upon activation, we next examined its nuclear localization as an indicator of the active FOS fraction. Nuclear FOS levels at 72 and 96 h were significantly higher under acidic conditions compared with neutral pH (Figure 2D, *p* = 0.0079 and *p* = 0.0119, respectively), consistent with the rise in *FOS* transcript levels. A transcriptomic analysis comparing 143B cells cultured under neutral or acidic conditions also revealed an increase in FOS transcript, significant in 2D (Figure 2E), and a large number of differentially expressed genes, including several directly connected to FOS (Figure 2F,G).

**FIGURE 3:**
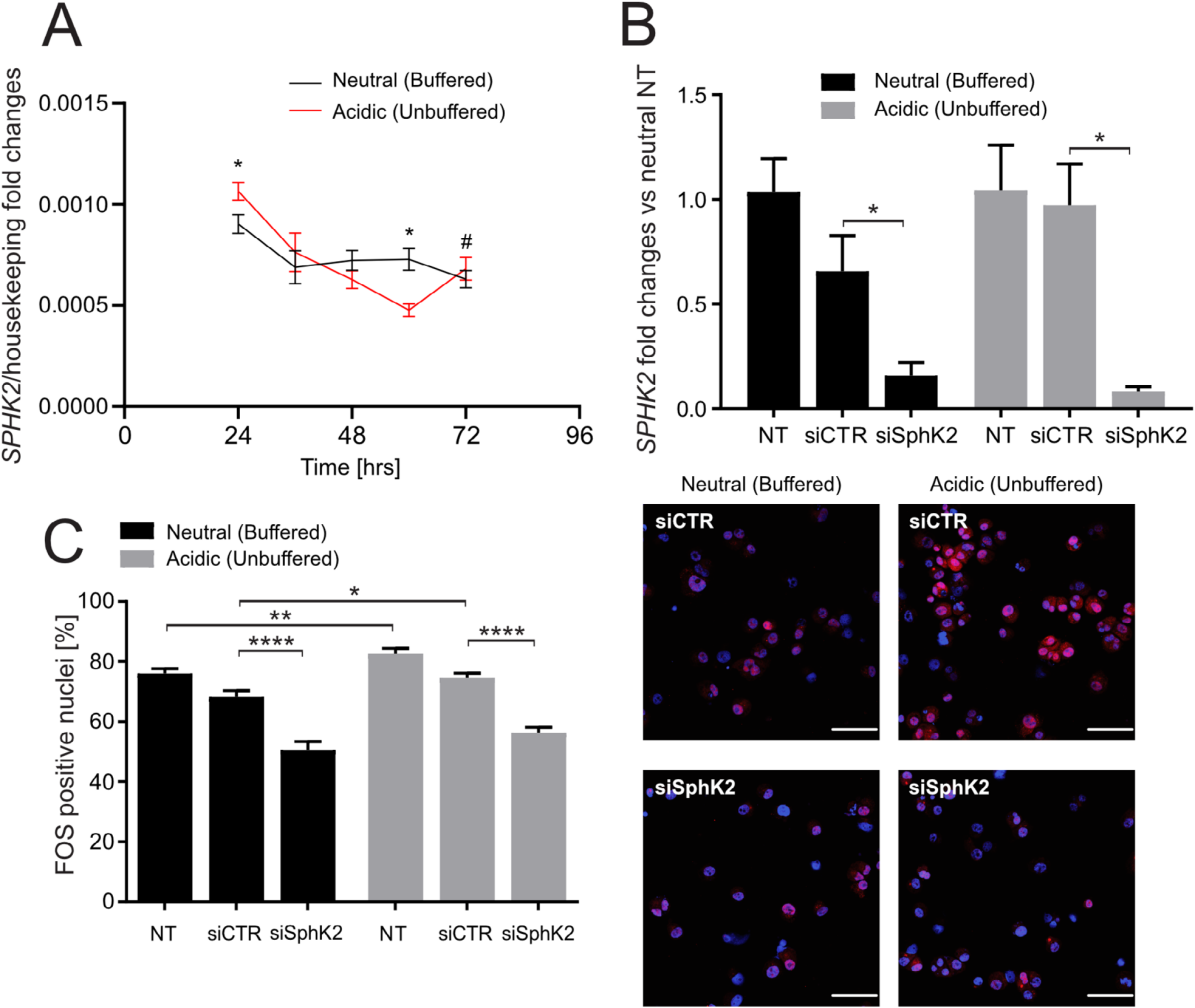
FOS upregulation in acidosis is dependent on SphK2 expression. (A) Q‐RT‐PCR of Sphk2 mRNA in 143B spheroids in neutral vs. acidic (unbuffered) medium at the indicated time points, normalized to three housekeeping genes (GAPDH, GUSB, YWHAZ) (*p < 0.05 at 24 h and 60 h between neutral and acidic, # *p* = 0.05 between acidic 60 h and acidic 72 h, *n* = 3). Results are expressed as fold changes in respect to the NT condition with neutral or acidic (unbuffered) medium. (B) Q‐RT‐PCR of SphK2 mRNA in 143B spheroids silenced with anti‐SphK2 siRNA in neutral vs. acidic (unbuffered) medium, normalized to three housekeeping genes (GAPDH, GUSB, YWHAZ). NT: Not electroporated; siCTR: Non‐targeting control pool siRNA; siSphK2: Anti‐SphK2 siRNA (*p < 0.05; *n* = 4); (C) Left: FOS positive nuclei (%) in 143B spheroids with anti‐SphK2 siRNA in neutral vs. acidic (unbuffered) medium by immunofluorescence (p** < 0.01; p**** < 0.0001, *n* = 12). Right: Representative images (red, nuclei were counterstained with bisBenzimide H 33258) (scale bar = 100 μm).

To clarify the contribution of nuclear S1P signaling under acidic stress, we performed a time‐course analysis of SphK2 expression, the kinase responsible for nuclear S1P synthesis. We found that acidosis induces a dynamic transcriptional response: SphK2 expression was significantly elevated at early time points, followed by a decline at later stages (Figure 3A; *p* = 0.0260 at 24 h), and a subsequent upward trend at 72 h. This transient pattern suggests that acidic stress promotes an early activation of nuclear S1P signaling that is not sustained over time, or that could be cyclically reactivated. Next, we used siRNA to silence SphK2. SphK2 knockdown significantly reduced its expression under both pH conditions, while non‐targeting siRNA (siCTR) had no effect (Figure 3B, *p* = 0.0286 for neutral and *p* = 0.0143 for acidic). As a result of the gene silencing, FOS nuclear localization was significantly decreased compared with non‐electroporated controls (NT) and siCTR‐treated cells, at the respective pH (Figure 3C, *p* < 0.0001 for both neutral and acidic), indicating that FOS activation depends on SphK2‐derived S1P irrespective of extracellular pH.

**FIGURE 4:**
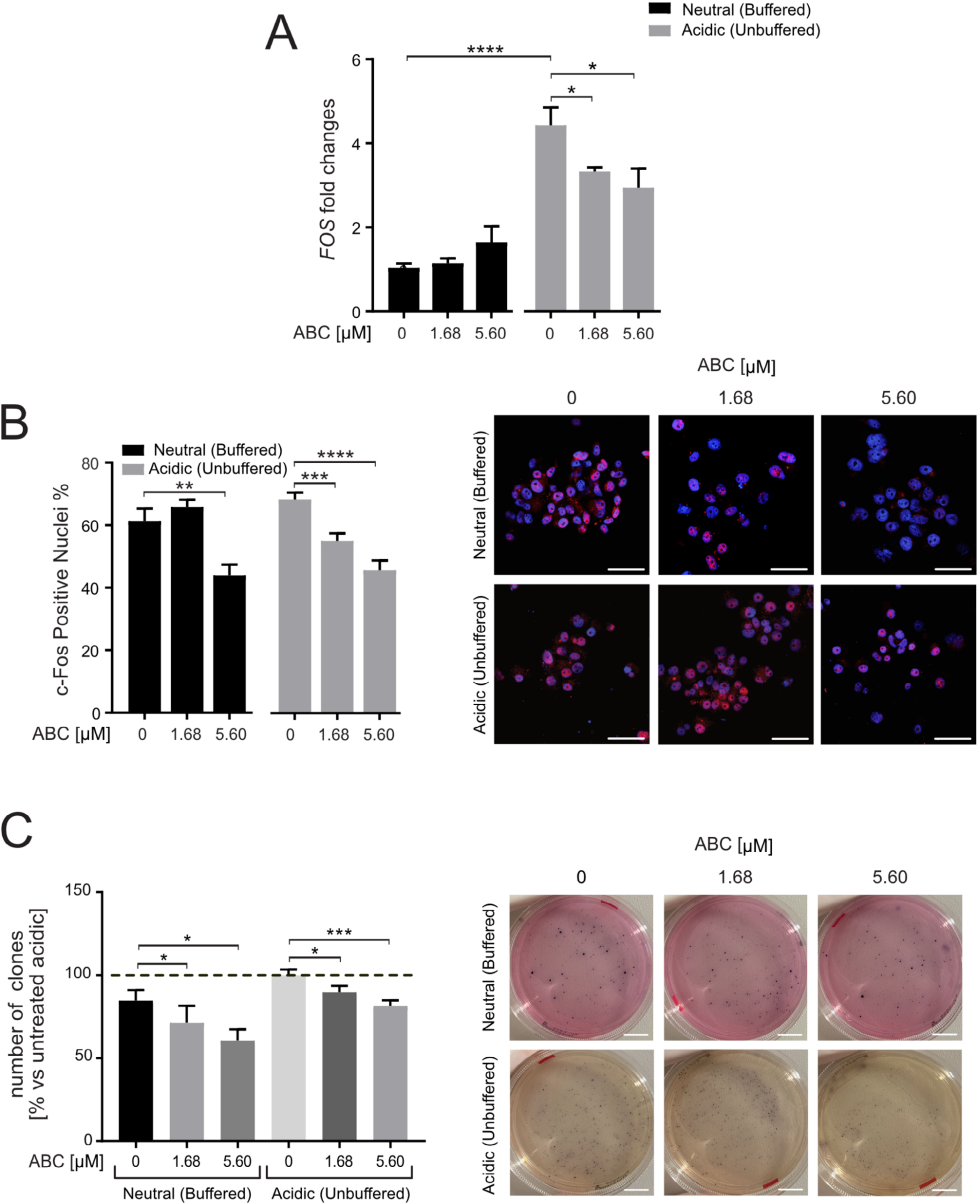
ABC294640 reduces FOS expression and nuclear localization, and clone formation in OS cells. (A) Q‐RT‐PCR of *FOS* mRNA in 143B spheroids in neutral vs. acidic (unbuffered) medium ± ABC294640, normalized to three housekeeping genes (GAPDH, GUSB, YWHAZ) (*p < 0.05, ***p < 0.001, *n* = 4); (B) Upper: Representative FOS immunofluorescence in 143B spheroids in neutral vs. acidic (unbuffered) medium ± ABC294640 at indicated concentrations (red, nuclei were counterstained with bisBenzimide H 33258) (scale bar 100 μm); Lower: FOS positive nuclei (%) (**p < 0.01, ***p < 0.001, ****p < 0.0001, *n* = 10); (C) Upper: Quantification and representative images of clonogenesis in soft‐agar assay in neutral vs. acidic (unbuffered) medium ±ABC294640, grown for 14 days before MTT staining (*p < 0.05, ***p < 0.001, *n* = 12, results expressed as percentage in respect to the acidic CTR value). One‐tailed Mann–Whitney test, mean ± SEM. Lower: Representative images of the clonogenesis assay in soft‐agar.

We further validated this mechanism pharmacologically using ABC294640, a selective SphK2 inhibitor. In 143B spheroids, ABC294640 reduced *FOS* mRNA expression, but only under acidic conditions (Figure 4A, *p* = 0.0190 for IC15 and *p* = 0.0190 for IC 50 and Figure S5). Conversely, MG‐63 spheroids showed a significant response only under neutral pH, while HOS do not show any significant change (Figure S6). At the protein level, ABC294640 caused a dose‐dependent reduction in nuclear FOS localization in 143B spheroids under both pH conditions (Figure 4B, *p* = 0.0039 for neutral CTR versus ABD294640 at IC 15; *p* = 0.0007 for acidic CTR versus ABC294640 at IC15, *p* < 0.0001 versus ABC294640 at IC50). Importantly, repeating the experiment in buffered pH 6.8 conditions (Supplementary Figure S7), confirmed that the effect of acidosis is reproducible across controlled acidic environments. Similar effects were observed in MG‐63 spheroids, whereas HOS spheroids showed no increase of FOS nuclearization in acidic conditions (Figure S6).

To assess the functional impact of SphK2‐dependent FOS activation, we performed a soft‐agar colony formation assay. OS cells cultured under acidic conditions formed fewer colonies than those in neutral pH (Figure 4C). Importantly, ABC294640 treatment further reduced colony formation in both environments, with a more pronounced effect observed under acidic conditions (Figure 4C), suggesting that SphK2 activity supports tumorigenic potential in this context, particularly when cells are exposed to an acidic microenvironment. Overall, our results indicate that blockage of SphK2 synthesis of nuclear S1P reduces *FOS* transcription specifically at low pH, possibly preventing S1P‐mediated HDAC inhibition. This finding further validates the strong connection between acidosis‐induced S1P and the activation of the *FOS* oncogene.

### 2.3 Acidic Microenvironment Drives SphK2 Upregulation and FOS Expression in OS Patient Tissues

**FIGURE 5:**
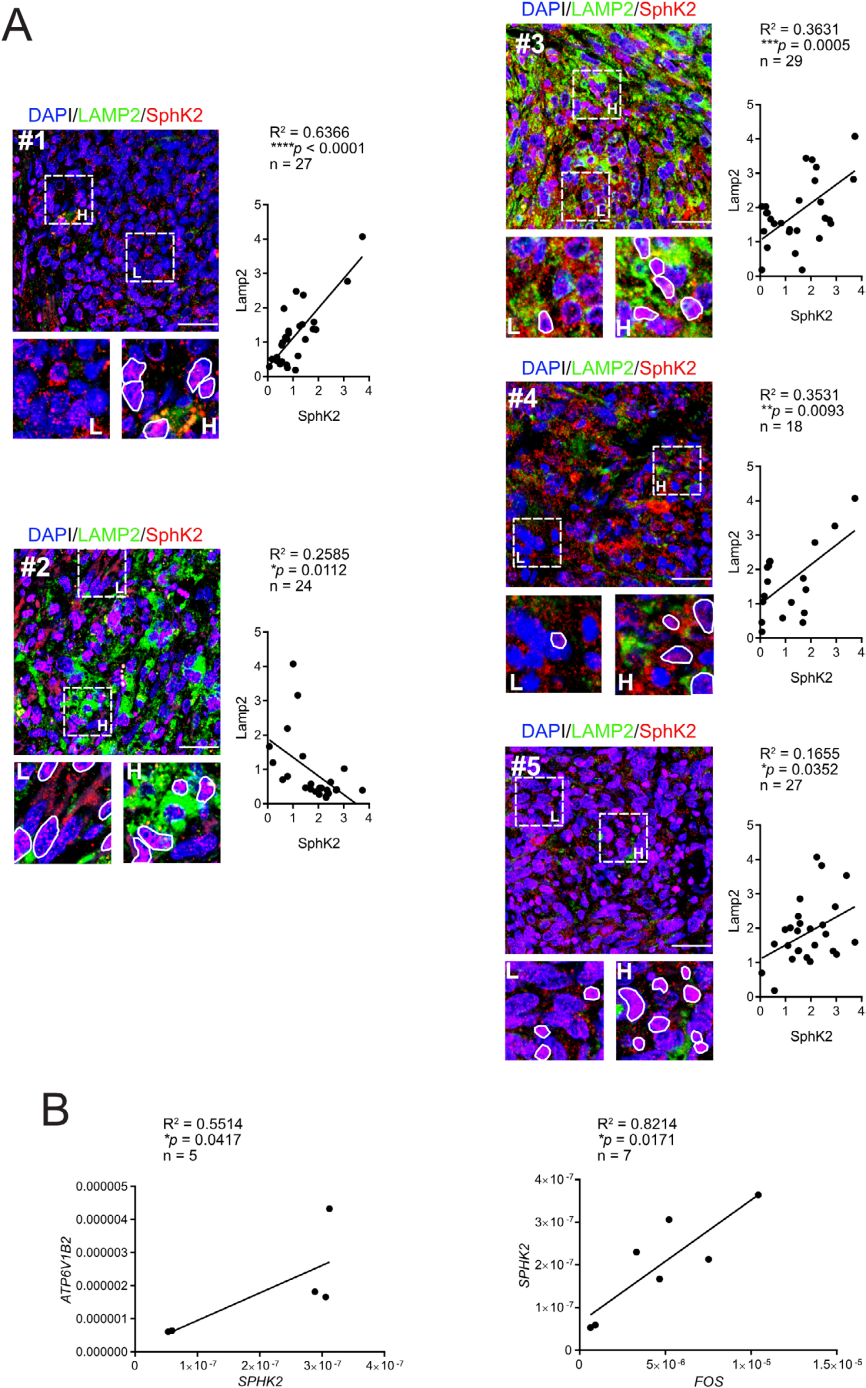
Acidosis promotes S1P‐mediated upregulation of FOS expression and nuclear localization in human osteosarcoma. (A) SphK2 and LAMP2 Immunofluorescence in lung metastases from 5 human osteosarcoma patients. Left: Representative images (scale bar 100 μm) with enlarged insets showing low and high LAMP2 signal areas, and contoured nuclei with ~ > 50% nuclear area positive for SphK2. Right: Quantification and correlation of normalized values (Pearson *r* test, *p < 0.05, **p < 0.01, ***p < 0.001, ****p < 0.0001); (B) Correlation of SphK2 mRNA levels with V‐ATPase and FOS in OS tissues from patients, normalized against three housekeeping genes (GAPDH, GUSB, YWHAZ) (one‐tailed Spearman Rank test, *p < 0.05).

To confirm the causal correlation of the acidic microenvironment and S1P accumulation in the TME, we analyzed the co‐localization of SphK2 and LAMP2, an indirect marker of extracellular acidosis [35], by immunofluorescence in tissue sections from lung metastases of human OS patients. Notably, we observed a strong and statistically significant positive correlation between SphK2 and LAMP2 in four out of five cases (Figure 5A).

We next evaluated SphK2 mRNA levels and their correlation with the expression of V‐ATPase V1B2, a subunit of the vacuolar proton pump and another established marker of acidosis [36, 37], in primary tumor tissues from patients with primary OS (Figure 5B). Our analysis revealed a significant positive correlation between SphK2 and V‐ATPase V1B2 expression. Most importantly, we also found a strong correlation between SphK2 and *FOS* mRNA levels (Figure 5B).

These findings provide further evidence associating extracellular acidosis with nuclear S1P accumulation, as indirectly demonstrated by SphK2 staining and FOS activation in OS.

## 3 Discussion

The TME is increasingly recognized as a key driver in cancer progression. Among its components, extracellular acidosis derived by the tumor cells themselves plays a central role in promoting tumor aggressiveness. Despite this, few studies have explored how changes in extracellular pH influence the activation of key oncogenes involved in cellular transformation [16, 38, 39, 40]. Recent evidence suggests that acidosis resulting from altered tumor metabolism induces widespread metabolic, genetic, and epigenetic changes. However, the underlying mechanism remains largely unclear [1, 2, 3]. In this study, we identify a rapid, indirect mechanism by which cancer cells respond to extracellular acidosis. This response is mediated by the nuclear accumulation of S1P, a bioactive sphingolipid known to support cell survival [25].

To this aim, we first verified whether elevated S1P in acidic TME could increase histone H3K9 acetylation, as previously reported for other stimuli [27, 28]. In acid‐exposed 143B cells, we observed a significant increase in H3 acetylation, supporting the hypothesis that extracellular acidosis promotes epigenetic remodeling in aggressive OS. In contrast, no increase in H3 acetylation was observed in acid‐exposed MG‐63 cells, which are less aggressive. This result aligns with previous studies showing that extracellular acidosis alters the acetylation profile of mesenchymal cells, but not of MG‐63 cells [9]. Interestingly, we could also observe a modest increase in H3 acetylation over time under neutral pH conditions; this likely reflects intrinsic time‐dependent adaptations associated with 3D spheroid growth rather than pH‐driven effects. Spheroid growth is known to induce progressive metabolic and transcriptional reprogramming, which can influence chromatin dynamics independently of extracellular acidity. Consistently, recent evidence indicates that both histone methylation and acetylation marks increase during the transition from 2D to 3D culture over time in non‐small cell lung cancer models [41, 42]. Thus, the acetylation changes observed under neutral conditions are likely attributable to 3D culture–driven epigenetic adaptation rather than to acidosis‐related mechanisms. Importantly, however, the magnitude and kinetics of acetylation under acidic conditions in 143B cells exceeded those observed in neutral controls, supporting the interpretation that acidosis acts as a specific amplifier of epigenetic remodeling rather than merely reflecting time‐dependent culture adaptation. Together, these findings support the hypothesis that acidosis can reshape the epigenetic landscape of OS through S1P accumulation, particularly in more aggressive tumor subtypes. Finally, extracellular acidosis has also been reported in carcinoma models to induce selective sirtuin‐mediated deacetylation programs, particularly involving SIRT1 and SIRT6, resulting in repression of metabolic targets such as acetyl‐CoA carboxylase 2 and consequent rewiring of lipid metabolism [12, 14, 43]. These findings suggest that acidosis does not uniformly increase acetylation but instead drives context‐dependent epigenetic remodeling. In this framework, sirtuin activation may promote metabolic adaptation through deacetylation, whereas nuclear S1P accumulation may favor localized histone acetylation linked to oncogenic transcription. The potential coexistence of these bidirectional regulatory mechanisms, balancing survival, energy homeostasis, and transcriptional plasticity under acidic stress, remains to be investigated in OS, where this interplay has not yet been thoroughly explored. However, it should be emphasized that this model remains hypothetical, as the tumor systems examined in the cited studies differ substantially from OS, and it cannot be assumed that these mechanisms necessarily coexist within the same biological context. Clarifying whether such regulatory programs operate in parallel or represent tumor type–specific adaptations will be an important objective for future investigations.

Overexpression of specific genes due to dysregulation of histone H3 acetylation has been associated with various tumor types [44, 45, 46, 47], including OS [48, 49]. One such gene, specifically induced by S1P‐mediated inhibition of HDACs, is FOS, a transcription factor and oncoprotein associated with OS cell differentiation, proliferation, and aggressiveness. FOS overexpression alone can drive OS tumorigenesis in a mouse model [32] and is frequently detected in relapses and metastases [31], making it a negative prognostic marker [50]. In line with this, our study revealed elevated *FOS* expression in metastatic 143B OS spheroids compared with normal osteoblasts. *FOS* levels were also higher in 3D spheroid cultures than in 2D monolayer, highlighting phenotypic differences between these models [51, 52, 53]. Given the established connection between acidic microenvironment, *FOS* expression, and metastasis [15, 22], we investigated whether the increased H3 acetylation observed under acidic conditions contributes to FOS activation. Indeed, both *FOS* gene expression and FOS nuclear localization were significantly elevated in 143B spheroids cultured in acidic conditions compared with neutral pH. Notably, the transient reduction in FOS activity observed in neutral conditions at 72 h likely reflects its previously reported biological regulation. Immediate‐early genes such as FOS are known to exhibit oscillatory and circadian‐like transcriptional dynamics during prolonged monitoring, which may produce temporary dips in expression despite sustained upstream signaling [54]. Furthermore, a transcriptomic analysis revealed several upregulated genes connected to the FOS pathway under acidic vs. neutral conditions that may play a role in OS. For example, *NFATc1* expression has been shown to be partially dependent on *FOS* expression [55]. *EGR1* is considered a prognostic gene for OS metastasis, alongside *FOS* [50]. Likewise, FOSL1 has been proven to facilitate OS metastasis [56] and shows co‐expression with FOS according to the STRING database [57]. Notably, FOSL1/Fra‐1 is a direct FOS‐target gene [58, 59]. However, increased FOS expression does not imply uniform activation of all FOS‐associated genes; acidic stress likely reshapes transcriptional networks, producing selective up‐ and downregulation of downstream targets depending on co‐factor context and competing signaling pathways. Consistent with this notion, a more detailed analysis of FOS staining in cytocentrifuged cells isolated from spheroids revealed heterogeneous nuclear localization within the same microscopic field. This non‐uniform pattern may reflect intrinsic cellular heterogeneity within 3D spheroids, leading to variable responses even among clonal populations, and/or the transient and tightly regulated nature of FOS nuclear localization, which is characteristic of many immediate‐early transcription factors.

Together, these findings confirm that acidosis promotes *FOS* via S1P accumulation, and that this also upregulates a number of *FOS‐related* genes. To determine whether this effect is specifically mediated by nuclear S1P through HDAC inhibition, rather than by cytosolic S1P signaling, we silenced SphK2 using siRNA. Notably, our time‐course analysis indicates that SphK2 expression responds dynamically to acidic stress, showing an early increase followed by a later decline. Notably, the extended time‐course analysis revealed a dynamic, oscillatory pattern of SphK2 expression under acidic conditions, with an initial peak at 24 h followed by a decline, and a second trend but significant increase at 72 h. This temporal profile is consistent with a sequential regulatory cascade in which SphK2 activity precedes S1P accumulation, histone acetylation, and subsequent FOS activation. In line with this model, the increase in nuclear FOS staining observed at 96 h occurred approximately 24 h after the second SphK2 peak, supporting a biologically coherent delay. Together, these findings further strengthen the interpretation that nuclear S1P signaling under acidic stress represents a dynamically regulated adaptive response. Together, these observations support the view that nuclear S1P signaling under acidic conditions reflects a dynamic adaptive response rather than a simple linear relationship with mRNA abundance. While cytosolic S1P has been demonstrated in other models to activate S1PR2 and enhance FOS expression [60], and also modulate the nuclear localization of other transcription factors [61, 62], our results point to a nuclear S1P‐specific mechanism. SphK2 knockdown significantly reduced FOS nuclear localization. Nevertheless, while our data support a nuclear S1P–dependent mechanism downstream of acidosis, it is plausible that extracellular acidification may also enhance S1P signaling indirectly through stress pathways such as endoplasmic reticulum stress, which has been reported to modulate sphingolipid metabolism [63, 64]. This suggests that multiple acid‐induced cellular responses may converge to reinforce S1P‐driven epigenetic regulation.

**FIGURE 6:**
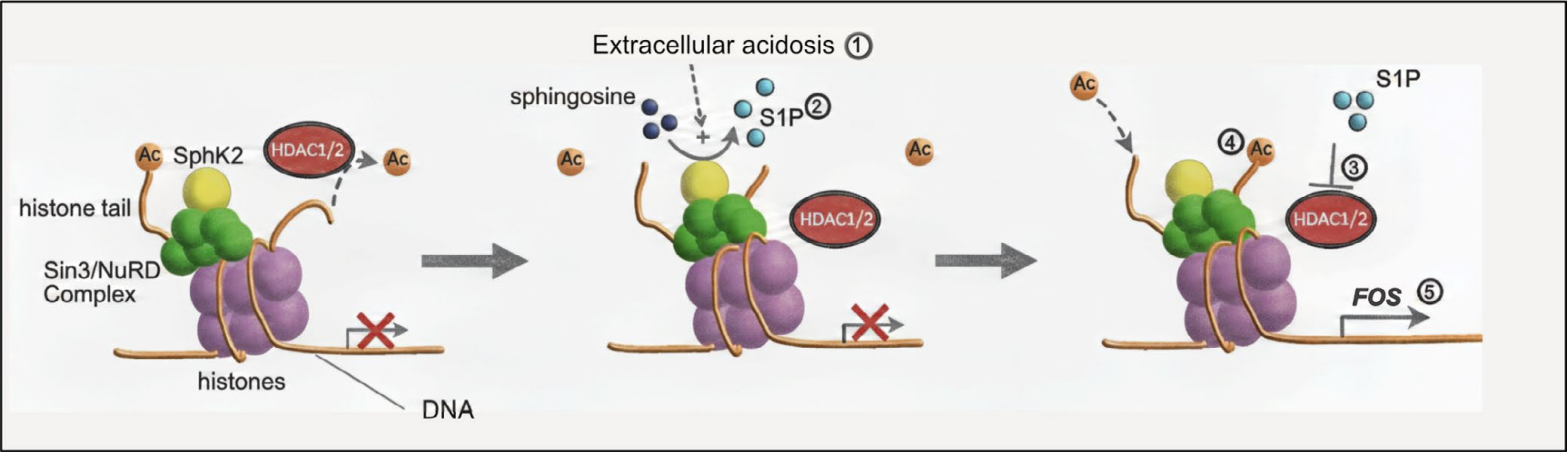
Proposed mechanism of FOS indirect activation by extracellular acidosis (adapted from Bozzini et al. Int J Mol Sci. 2023;24:5294).

In summary, based on the results in vitro, the proposed mechanistic model of acidosis‐driven FOS activation is (Figure 6): in untreated conditions, HDAC deacetylates histones, decreasing gene expression; [1] the formation of extracellular acidosis indirectly promotes SphK2 localization in the nucleus of tumor cells, [2], which synthesizes S1P at inhibitory complexes for gene expression; [3] S1P inhibits HDAC, [4] leading to increased acetylation and [5] expression of *FOS*.

This may represent a key homeostatic mechanism that allows OS cells to maintain their epigenetic profile, evade immune surveillance, and endure in hostile metastatic environment [5, 40]. Our findings highlight the importance and therapeutic potential of targeting this regulatory axis in acidic tumors such as OS. Several therapies targeting acidosis are currently in development [10]. In this study, we evaluated ABC294640, a selective and orally available SphK2 inhibitor, for its potential to counteract OS aggressiveness under acidic conditions. This drug has already been evaluated for cancer treatment [65, 66, 67, 68, 69, 70] and is currently undergoing clinical trials [71]. However, its efficacy against acid‐adapted tumors remains largely unexplored. We found that ABC294640, similar to SphK2 silencing, significantly reduced FOS expression and nuclear localization in 143B OS cells, likely by disrupting S1P‐mediated inhibition of HDAC1/2. In contrast, the response was attenuated in less aggressive models: MG‐63 cells showed a significant reduction in FOS expression only under neutral conditions, while HOS cells exhibited no measurable change following treatment. This differential sensitivity is consistent with the biological hierarchy of these models, as highly metastatic 143B cells are known to display enhanced stress‐adaptive and oncogenic signaling compared with their nonmetastatic counterparts [72, 73]. The absence or attenuation of the phenotype in MG‐63 and HOS cells therefore likely reflects their reduced reliance on S1P–FOS signaling to sustain aggressive behavior, rather than a lack of drug activity per se. While this association does not establish causality, it supports the interpretation that the S1P–FOS axis is preferentially engaged in metastatic OS phenotypes.

Additionally, we looked to the ABC294640 effects on OS tumorigenesis in vitro, by soft‐agar assay. OS cells cultured under neutral conditions formed fewer, but larger colonies than those in acidic pH, consistent with the trend that acidosis promotes aggressiveness and dissemination. Furthermore, though ABC294640 treatment decreased the formation of OS clones in both conditions, the effect was more pronounced under acidosis, highlighting the stronger reliance of acidic‐cultured OS cells on the S1P‐FOS axis.

Finally, to determine whether the proposed mechanism is confirmed in patient tumors, we analyzed the expression levels and spatial co‐localization of key proteins involved in this pathway in both primary and metastatic OS tissue samples. The results from patient samples were consistent with our in vitro findings.

Specifically, we observed a positive correlation between SphK2 and V‐ATPase mRNA levels—V‐ATPase being a known marker of extracellular acidity [74]. In addition, we detected nuclear localization of SphK2 in tumor cells, which was frequently accompanied by elevated LAMP2 expression in the surrounding regions. Like V‐ATPase, LAMP2 serves as an indirect protein‐level marker of acidity [35] in tumor tissues from OS patients, further supporting a link between nuclear S1P signaling and the acidic tumor microenvironment. Notably, the association between LAMP2 and acidosis has been linked to its relocalization to the plasma membrane [35]. While our analysis confirms increased LAMP2 expression in acidic tumor regions, it does not allow definitive conclusions regarding its precise subcellular distribution. Future studies specifically addressing membrane relocalization of LAMP2 in OS would therefore be of considerable interest. We also found a significant positive correlation between SphK2 and *FOS* mRNA expression levels in OS tumor samples, reinforcing the connection between SphK2 activity and FOS–mediated oncogenic signaling in acidic conditions. These findings tie everything together, confirming the proposed mechanism.

## 4 Materials and Methods

### 4.1 Cell Cultures and Reagents

All cell lines were purchased from the American Type Culture Collection (ATCC, Manassas, Virginia, USA) and cultured in Iscove's Modified Dulbecco's Medium (IMDM, Thermo Fisher Scientific, Waltham, Massachusetts, USA) enriched with 10% heat‐inactivated fetal bovine serum (Euroclone, Milan, Italy) and 1% Penicillin/Streptomycin Solution (Euroclone). Cells were grown at 37°C in a humidified atmosphere with 5% CO_2_. Cells were tested against mycoplasma using bisBenzimide H 33258 coloration (Sigma‐Aldrich, St. Louis, Missouri, USA). Cells were validated (LGC Standards, Milan, Italy) in 2023.

To generate 3D spheroids, 5 × 10^3^ cells for the 143B cell line and 6 × 10^3^ for the MG‐63 cells were plated in PrimeSurface 3D Culture Spheroid plates: Ultra‐low Attachment (ULA) Plates (MS‐9096UZ, S‐bio, Hudson, New Hampshire, USA) in 200 μL of filtered RPMI medium (Sigma‐Aldrich). RPMI (R6504, Sigma‐Aldrich) was either buffered with the addition of 2 g/L sodium bicarbonate during powder dissolution to generate pH = 7.4, or left unbuffered, meaning pure, bicarbonate‐free medium, allowing the balance with the external atmosphere and the downstream effects of the metabolic activity of spheroids to change medium pH. Buffered pH 6.8 medium was obtained by the supplementation with sodium bicarbonate in the amount calculated by the Henderson‐Hasselbach equation. For treatment experiments, spheroids were treated with 5.6 μM (IC50) or 1.7 μM (IC15) ABC294640 (A110039, AmBeed, Arlington Heights, Illinois, USA) (Figure S5A). IC15 and IC50 were assessed as cell mortality rate over logarithm of treatment concentration, Figure S5. Spheroids were grown for up to 96 h.

### 4.2 Gene Silencing via siRNA

Silencing of specific genes was achieved through siRNA delivered by pipette‐type electroporation. 143B cells were detached with trypsin and counted after staining with erythrosine dye. 0.2 nmol of ONTARGETplus siRNA SMARTpool (Dharmacon, Lafayette, Colorado, USA) against SphK2, or ONTARGETplus non‐targeting control pool (Dharmacon), were added to a 15‐μL suspension containing 300 000 cells. 10 μL of suspension were aspirated with a 1‐mm capillary tip (Neon Transfection System, Invitrogen, Life Technologies), and the tip was then docked into a microporator (Digital Bio, Watertown, Massachusetts). Cells were electroporated at 1250 V for 10 ms, in 3 pulses. After electroporation, cells were resuspended in pen/strep‐free medium at pH 7.4 or unbuffered and seeded in 96‐well plates (200 μL/well, 5000 cells/well) for either RNA extraction or cytocentrifugation. After 24 h, culture medium was changed to a complete medium at pH 7.4 or unbuffered. For S1P treatment, 1 μM S1P (Sigma‐Aldrich) was added each day. Cells were grown after electroporation for 48 h for RNA extraction or 72 h for cytocentrifugation.

### 4.3 RNA Isolation, Gene Expression and RNA‐Seq

RNA for real‐time PCR assays was extracted using TRIzol Reagent (Thermo Fisher Scientific), and retrotranscription was performed using MultiScribe Reverse Transcriptase (Thermo Fisher Scientific). Real‐Time polymerization chain reaction (real‐time PCR) was performed by amplifying 1 μg of cDNA using SsoAdvanced Universal SYBR Green Supermix (BioRad, Hercules, California, USA) and the CFX96Touch instrument (BioRad). Primers were generated via a web‐based primer design software (https://primer3.ut.ee/). Three different housekeeping genes were used for normalization: *glyceraldehyde‐3‐phosphate dehydrogenase* (GAPDH), *glucoronidase beta* (GUSB), and *Tyrosine 3‐Monooxygenase/Tryptophan 5‐Monooxygenase Activation Protein Zeta* (*YWHAZ*). This was done to compensate for inconsistent expression under acidic conditions [75]. Primer sequences were as follows: *GAPDH* For: ccaaggagtaagacccctgg; *GAPDH* Rev.: aggggagattcagtgtggtg; *GUSB* For: cccactcagtagccaagtca; *GUSB* Rev.: gttctgctgctgtggaagtc; *YWHAZ* For: ccgcatgatctttctggctc; *YWHAZ* Rev.: tagtctgtgggatgcaagca; mV‐ATPase *V1B2* For: tggccgaagacttccttg; *V‐ATPase V1B2* Rev.: ccgaaatgccagtctgaatc; *PLIN2* For: tcagctccattctactgttcacc; *PLIN2* Rev.: cctgaattttctgattggcact; *FOS* For: gcttcaacgcagactacgag; *FOS* Rev.: tgcgggtgagtggtagtaag; *SPHK2* For: ctgagtgagtgggatggcat; *SPHK2* Rev.: aggcatcttcacagcttcct.

RNA for RNA‐Seq was extracted using RNeasy Mini Kit (74 104, QIAGEN, Hilden, Germany), following the instructions provided in the kit. RNA‐seq libraries were obtained starting from 100 ng of total RNA following Illumina Stranded TotalRNA Prep Ligation with Ribo‐zero Plus protocol. Sequencing data quality was assessed using the FastQC v0.11.9 software. High‐quality paired‐end reads were aligned to the human reference genome (GRCh38 p14) and genes were annotated on Gencode v47. RSEM v1.3.1 was applied on paired FASTQ files to map counts through STAR (v2.7.11a) and estimate transcript abundance. Differential analysis was performed with the R package DESeq2 v1.32.0.

### 4.4 Capillary Western Blotting

To extract proteins, cell spheroids were harvested and lysed using hot RIPA buffer (50 mM Tris–HCl, 150 mM NaCL, 1% Triton X‐100, 0.5% Sodium deoxycholate, 2% SDS, 0.5 mM EGTA and 10 mM NaF). Protein concentrations were analyzed using BCA Protein Assay Kit (Thermo Fisher Scientific). All Western Blots were performed using Simple Western Automated Western Blot System (Abby, Bio‐Techne, Minneapolis, Minnesota, USA) and following the instructions provided by the manufacturer. Specific protein quantification was performed automatically using the Compass software for Simple Western (version 6.2.0, Bio‐Techne, https://www.bio‐techne.com/resources/instrument‐software‐download‐center/compass‐software‐simple‐western). Antibody signal was normalized on Total Protein signal (Total Protein Assay). Specifically, one capillary was selected as the Total Protein reference value (TPR), with the signal of all other capillaries being multiplied by a factor of $\frac{\text{Capillary Total Protein}}{\mathrm{TPR}}$. Virtual gel blots showed in this study were generated automatically by the software for visualization.

The primary antibodies and concentrations utilized were as follows: anti‐histone H3 1:50 (4499S, Cell Signaling Technology, Danvers, Massachusetts, USA); anti‐acetylated histone H3 1:50 (06–599, Millipore, Burlington, Massachusetts, USA); anti‐FOS 1:25 (pc05, Calbiochem, San Diego, California, USA).

### 4.5 Immunofluorescence

For immunofluorescence on 3D cells, spheroids were harvested, disrupted via up‐down pipetting for 143B and MG‐63 and trypsinization for HOS spheroids, and transferred on a glass slide (Epredia Polysine Adhesion Slides, J2800AMNZ, Thermo Fisher Scientific) using a Cyto‐Tek centrifuge (Electron Microscopy Science, Hatfield, Pennsylvania, USA) at 800 rpm for 5 min. The cells were then fixed with 3.7% paraformaldehyde for 20 min. Slides were incubated with anti‐FOS 1:25 (pc05, Calbiochem), anti‐FOS 1:25 (MA121190, Thermo Fisher Scientific), anti‐SphK2 1:50 (ab37977, Abcam, Cambridge, UK), or anti‐Lamp2 1:400 (HPA029100, Sigma‐Aldrich) overnight in a humid environment, followed by secondary Alexa Fluor 568 1:500, Alexa Fluor 488 1:500, or Alexa Fluor 647 1:250 (Life Technologies, Carlsbad, California, USA) for 30 min. Nuclei were counterstained with 2.25 g/mL of bisBenzimide H 33258 (Sigma‐Aldrich). Images were acquired by confocal microscopy (AiR MP confocal microscope, Nikon, Minato, Tokyo, Japan), using the 20× elevated‐working distance objective, Galvano scanning, zoom set to 1, 0.6 numerical aperture, and line average of 4.

For immunofluorescence on patient‐derived tumor sections, full protocol for slide preparation can be found in Supporting Information. In brief, after dewaxing in Histoclear (R0050CITRO, Histo Line Laboratories, Milan, Italy), samples were incubated in blocking solution for 1 h. Sections were then incubated overnight with anti‐SphK2 1:50 (ab37977, Abcam) and Alexa‐488‐conjugated anti‐Lamp2 1:100 (ab187607, Abcam), then with secondary antibody (Alexa Fluor 568 goat anti‐rabbit IgG 1:400, A11011, Invitrogen, Life Technologies). Sections were treated for autofluorescence quenching using Vector TrueView Autofluorescence kit with DAPI (SP‐8500‐15, VectorLabs, Newark, California, USA), following the kit instructions, then mounted with the DAPI mounting agent included in the kit. Images were acquired by confocal microscopy (AiR MP confocal microscope, Nikon), using the 20× Plan Apo λ objective (NA 0.75, RI 1.0), Galvano scanning, zoom set to 3.497, Ex 402.7/487.2/561.3, Em 450.00/525.0/595.0, pinhole size 43.42. Images were analyzed using NIS‐Elements Advanced Research software (Nikon). For each image, three random 300 × 300 px regions were selected for quantification. The software's automated signal detection identified nuclei by detecting the DAPI signal. Manual adjustments were made when necessary. Nuclei were counted. The same detection method was then applied within the identified nuclei subregions (ROIs) to detect the SphK2 signal (568). The LAMP2 signal (488) was measured across the entire selected region. SphK2 signal intensity was normalized to the number of nuclei in each region. Data were first normalized within each patient using min‐max normalization. Then, inter‐patient normalization was performed using Z‐score normalization [76].

### 4.6 Soft‐Agar Assay

We used soft‐agar assay to assess OS tumorigenesis. 143B cells were seeded in 30 mm petri dishes coated with two layers of agarose gel: 2% agarose was dissolved in RPMI medium (pH 7.4 or unbuffered) to a working concentration of 0.4% agarose (bottom layer) or 0.2% agarose (top layer). 143B cells were added to the top layer solution. Petri dishes were then coated with 4 mL bottom layer solution, left to harden at 4°C for 5 min, and transferred to a stable environment at 37°C and 5% CO_2_ for 1 h. Then 1.5 mL of the cell‐enriched top layer solution were dispensed to each petri. We utilized two seeding densities of 1000 and 3300 cells/dish. The dishes were then left to solidify for 20 min at 4°C, then incubated at 37°C and 5% CO_2_. The following day, and once per week thereafter, 500 μL of culture medium were added to each dish. For treatments, 5.6 μM ABC294640 (A110039, AmBeed) was mixed into the added medium. After 2 weeks, cell clones were colored with 0.5 mg/mL Thiazolyl Blue Tetrazolium Bromide (Sigma‐Aldrich) in 500‐μL RPMI medium and counted. Results were expressed as percentage of clones in respect to the n. of clones observed at acidic controls.

### 4.7 Statistical Analysis

Quantitative results were expressed as arithmetic mean plus or minus the standard error of the mean (SEM). Due to the small number of observations, data were not considered as normally distributed and non‐parametric tests were used. Each dataset was tested for significant differences using one‐tailed Mann–Whitney exact test, or for correlation using the Spearman Rank test (one‐tailed), with *p* < 0.05 being considered as statistically significant. For immunofluorescence on patient‐derived tissue, significant correlation was tested using Pearson correlation coefficient. For transcriptomic analysis, data was expressed as‐log_10_ of adjusted *p*‐value, log_2_ of fold change, and arithmetic mean of gene counts. Statistical analysis was performed using R Statistical Software (v4.1.2; R Core Team 2021) with the DESeq2 package [77]. Graphs were produced using the ggplot2 package (https://ggplot2.tidyverse.org). All the other statistical analyses were performed using Prism 7.04 software (Graph‐Pad, San Diego, California, USA).

## 5 Conclusions

Our study uncovers a previously uncharacterized mechanism by which OS cells adapt to extracellular acidosis through nuclear S1P signaling. We demonstrate that acidosis promotes the nuclear localization of SphK2, leading to S1P‐mediated inhibition of HDAC1/2, increased histone acetylation, and upregulation of the oncogene *FOS*. This epigenetic adaptation appears to support tumor cell survival and aggressiveness in acidic microenvironments. Importantly, we show that pharmacological inhibition of SphK2 with ABC294640 disrupts this pathway, reducing FOS activation and impairing OS clonogenicity under acidic conditions. These findings not only validate the functional relevance of this mechanism in patient‐derived OS tissues but also highlight the therapeutic potential of targeting nuclear S1P signaling in acid‐adapted tumors. As acidosis is a hallmark of many solid tumors, this regulatory axis may represent a broader vulnerability across multiple cancer types and warrants further investigation in clinical settings.

## Author Contributions

**Sofia Avnet:** conceptualization, methodology, supervision, project administration, visualization, writing – original draft, writing – review and editing, formal analysis, validation, investigation, data curation. **Nicolò Bozzini:** methodology, data curation, investigation, validation, visualization, writing – original draft, formal analysis, writing – review and editing. **Margherita Cortini:** data curation, validation, visualization, writing – original draft, supervision, methodology, formal analysis, writing – review and editing. **Nicola Baldini:** conceptualization, funding acquisition, project administration, writing – review and editing. **Michael Dack:** formal analysis, investigation. **Veronica Manicardi:** data curation, methodology, investigation, resources, formal analysis. **Agamemnon E. Grigoriadis:** formal analysis.

## Funding

The research leading to these results has received funding from AIRC under IG 2018—ID. 21 403 project and the National Recovery and Resilience Plan, PNRRM4C2‐Investimento 1.4‐CN00000041, CUP B93D21010860004, funded by the European Union—NextGenerationEU “National Center for Gene Therapy and Drugs based on RNA Technology” (to Nicola Baldini).

## Ethics Statement

Tumor tissues for RNA extraction were collected after the institutional ethical committee approval (local Ethics Committee of Istituto Ortopedico Rizzoli, No. of approval 0033626). Tumor tissues for IF staining were collected after the institutional ethical committee approval (local Ethics Committee of Istituto Ortopedico Rizzoli), CE AVEC: 478/2024/Sper/IOR, No. of approval 0013925, 24/09/2024.

## Consent

Written and informed consent was obtained from all patients or their guardians.

## Conflicts of Interest

The authors declare no conflicts of interest.

## Supporting information


**Data S1:** Immunofluorescence staining of paraffin‐embedded tissue section.
**Figure S1:**. Quantification of the pH of the spheroids over time. The spheroids were cultured in neutral (buffered, pH 7.4), acidic (buffered, pH 6.8), and acidic (unbuffered) conditions at the indicated time points. Mean ± SEM. (*p < 0.05; **p < 0.01, *n* = 6).
**Figure S2:** Representative electropherograms of all capillary western blot assays included in this study. (A) Samples shown in Figure 1A; (B) samples shown in Figure 2B.
**Figure S3:** Acidosis increases acetylation in acidic (buffered, pH 6.8). Left panel, acetylated/non‐acetylated H3 ratio quantification (*p < 0.05, *n* = 6) of H3 and H3‐Ac in 143B spheroids under neutral vs. acidic conditions (buffered pH 6.8); right panel, representative images. One‐tailed Mann–Whitney test, mean ± SEM.
**Figure S4:** Acetylation of H3 does not change in MG‐63 spheroids. Upper: acetylated/non‐acetylated H3 ratio quantification after 72 h of exposure to acidic (unbuffered) conditions of MG‐63, as revealed by the representative capillary western blot. Mean ± SEM.
**Figure S5:** IC50 for ABC294640, assessed as 5,6 μM. Nonlinear fit of cell mortality rate over logarithm of treatment concentration.
**Figure S6:** Effect of ABC294640 and DMS treatment on FOS expression as revealed by real‐time PCR (A) and FOS nuclear localization as revealed by immunofluorescence (B) in MG‐63 and HOS cell lines. Mean ± SEM, (*p < 0.05; **p < 0.01, ***p < 0.001; ****p < 0.0001 *n* = 8).
**Figure S7:** ABC294640 reduces FOS expression and nuclear localization in acidic (buffered, pH 6.8) conditions. Top: representative FOS immunofluorescence in 143B spheroids in neutral vs. unbuffered medium over time by immunofluorescence (red, nuclei were counterstained with bisBenzimide H33258) (scale bar 100 μm); right: FOS positive nuclei (%) (**p < 0.01, ****p < 0.0001 *n* = 5).

## Data Availability

The data that support the findings of this study are openly available in Experimental Data at https://doi.org/10.6084/m9.figshare.30198253, reference number 30198253.
