# Supplementary material for: Sphingosine‐1‐Phosphate Promotes FOS Activation in Osteosarcoma Under Tumor Acidosis
Source: Acta Physiol (Oxf). 2026 Apr 26;242:e70214. doi: 10.1111/apha.70214 (PMC13111787; doi:10.1111/apha.70214)
Supplement: Supplementary file 1 — Data S1: Immunofluorescence staining of paraffin‐embedded tissue section. Figure S1:. Quantification of the pH of the spheroids over time. The spheroids were cultured in neutral (buffered, pH 7.4), acidic (buffered, pH 6.8), and acidic (unbuffered) conditions at the indicated time points. Mean ± SEM. (*p < 0.05; **p < 0.01, n = 6). Figure S2: Representative electropherograms of all capillary western blot assays included in this study. (A) Samples shown in Figure 1A; (B) samples shown in Figure 2B. Figure S3: Acidosis increases acetylation in acidic (buffered, pH 6.8). Left panel, acetylated/non‐acetylated H3 ratio quantification (*p < 0.05, n = 6) of H3 and H3‐Ac in 143B spheroids under neutral vs. acidic conditions (buffered pH 6.8); right panel, representative images. One‐tailed Mann–Whitney test, mean ± SEM. Figure S4: Acetylation of H3 does not change in MG‐63 spheroids. Upper: acetylated/non‐acetylated H3 ratio quantification after 72 h of exposure to acidic (unbuffered) conditions of MG‐63, as revealed by the representative capillary western blot. Mean ± SEM. Figure S5: IC50 for ABC294640, assessed as 5,6 μM. Nonlinear fit of cell mortality rate over logarithm of treatment concentration. Figure S6: Effect of ABC294640 and DMS treatment on FOS expression as revealed by real‐time PCR (A) and FOS nuclear localization as revealed by immunofluorescence (B) in MG‐63 and HOS cell lines. Mean ± SEM, (*p < 0.05; **p < 0.01, ***p < 0.001; ****p < 0.0001 n = 8). Figure S7: ABC294640 reduces FOS expression and nuclear localization in acidic (buffered, pH 6.8) conditions. Top: representative FOS immunofluorescence in 143B spheroids in neutral vs. unbuffered medium over time by immunofluorescence (red, nuclei were counterstained with bisBenzimide H33258) (scale bar 100 μm); right: FOS positive nuclei (%) (**p < 0.01, ****p < 0.0001 n = 5). [file APHA-242-e70214-s001.docx]

Index

[Supplementary Methods 2](#_Toc208958624)

[Immunofluorescence staining of paraffin-embedded tissue section 2](#_Toc208958625)

[Supplementary Figures 4](#_Toc208958626)

[**Suppl. Fig. 1.** 4](#_Toc208958627)

[**Suppl. Fig. 2** 5](#_Toc208958628)

[**Suppl. Fig. 3.** 6](#_Toc208958629)

[**Suppl. Fig. 4.** 7](#_Toc208958630)

[**Suppl. Fig. 5.** 8](#_Toc208958631)

[**Suppl. Fig. 6.** 9](#_Toc208958632)

[**Suppl. Fig. 7.** 10](#_Toc208958632)

# **Supplementary Methods**

## Immunofluorescence staining of paraffin-embedded tissue section

The slides were prepared as follows:

1. Paraffin was removed by submerging the slides in two containers filled with Citro Histoclear (R0050CITRO, Histo Line Laboratories, Milan, Italy) for 10 min and 20 min, respectively;
2. Sections were hydrated by submersion in decreasing concentrations of ethanol in distilled water, specifically 100-80-70-50%, for 2 min each, followed by pure distilled water;
3. Sections were circled with a hydrophobic marker (IHC PAP (mini) pen, ADI-950-232-00001, Enzo Life Sciences, Farmingdale, New York, USA), then incubated with a blocking solution of 10% BSA (Albumin Bovine, A-9306, Sigma-Aldrich) and 0.2% Triton X-100 in TBS for 1 hr;
4. After removal of previous solution, samples were washed in TBST three times, for 5 min each;
5. Sections were incubated overnight with anti-SphK2 1:50 (ab37977, Abcam) and Alexa-488-conjugated anti-Lamp2 1:100 (ab187607, Abcam) diluted in blocking solution, in a humid environment;
6. After removal of previous solution, samples were washed in TBST three times, for 10 min each;
7. Sections were incubated for 1 hrs in secondary antibody (Alexa Fluor 568 goat anti-rabbig IgG (H+L), A11011, Invitrogen, Life Technologies), in a dark and humid environment;
8. After removal of previous solution, samples were washed in TBST three times, for 10 min each, in a dark environment;
9. Sections were treated for autofluorescence quenching using Vector TrueView Autofluorescence kit with DAPI (SP-8500-15, VectorLabs, Newark, California, USA), following the kit instructions. Afterwards, two drops of the mounting agent with DAPI included in the kit were dispensed to each section, and the section was then covered with a coverslide. Slides were left in the dark at room temperature for 1 hrs in order for the mounting agent to dry, then stored at +4 °C before confocal analysis.

# **Supplementary Figures**


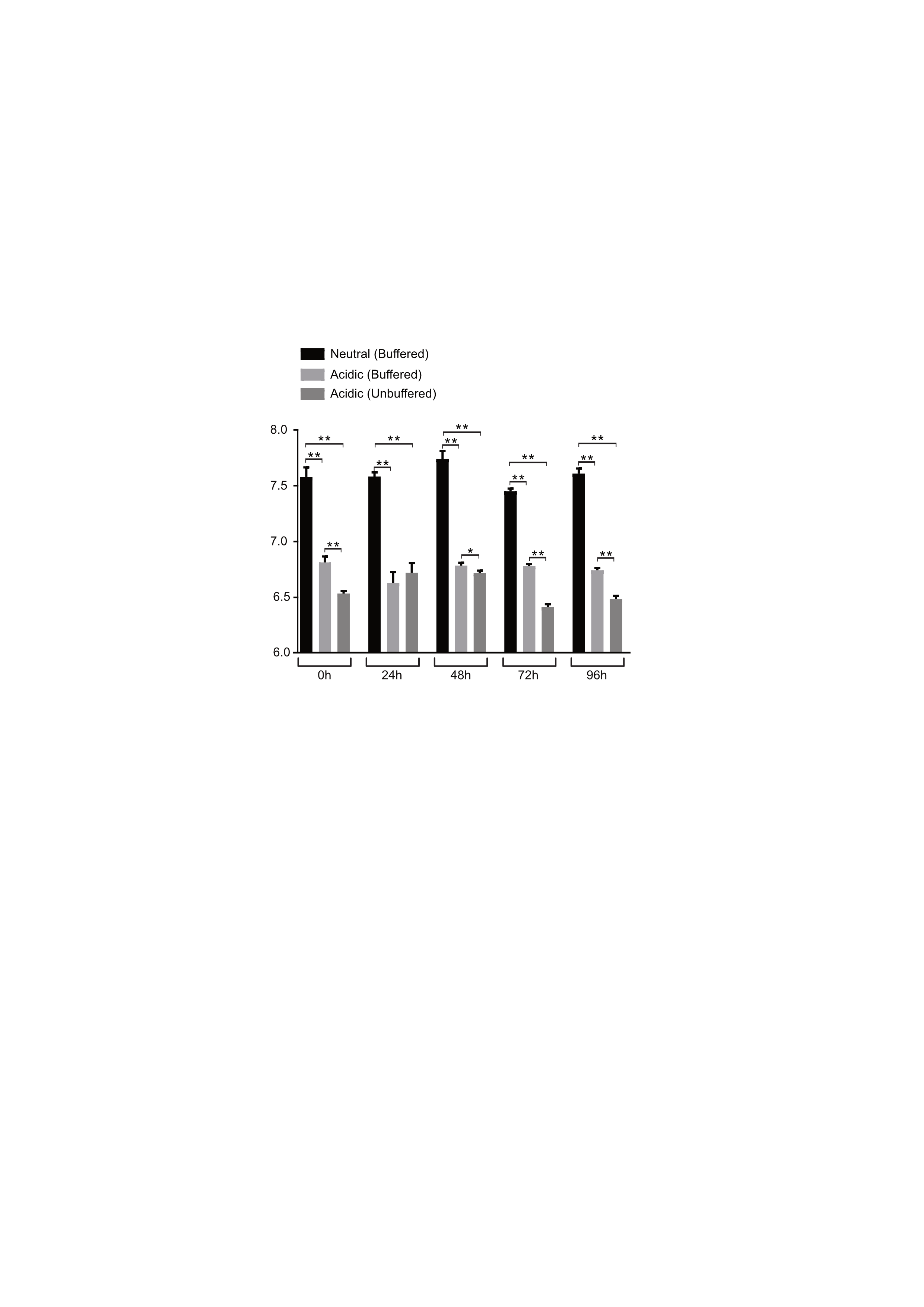


## **Suppl. Fig. 1.**

Quantification of the pH of the spheroids over time. The spheroids were cultured in neutral (buffered, pH 7.4), acidic (buffered, pH 6.8) and acidic (unbuffered) conditions at the indicated time points. Mean±SEM. (*p<0.05; **p<0.01, n=6).


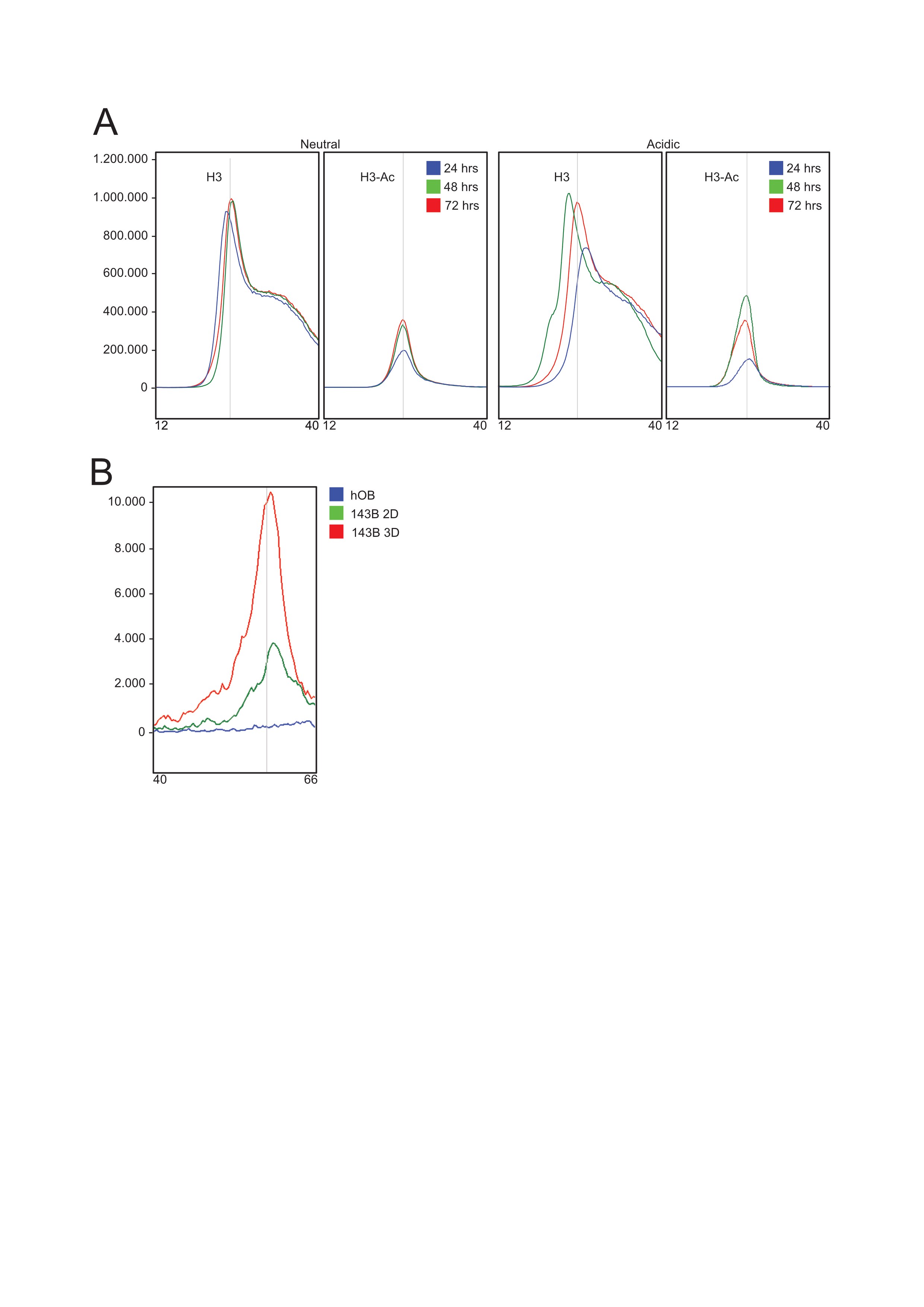


## **Suppl. Fig. 2.**

Representative electropherograms of all capillary western blot assays included in this study. **A**) samples showed in Fig. 1A; **B**) samples showed in Fig. 2B.


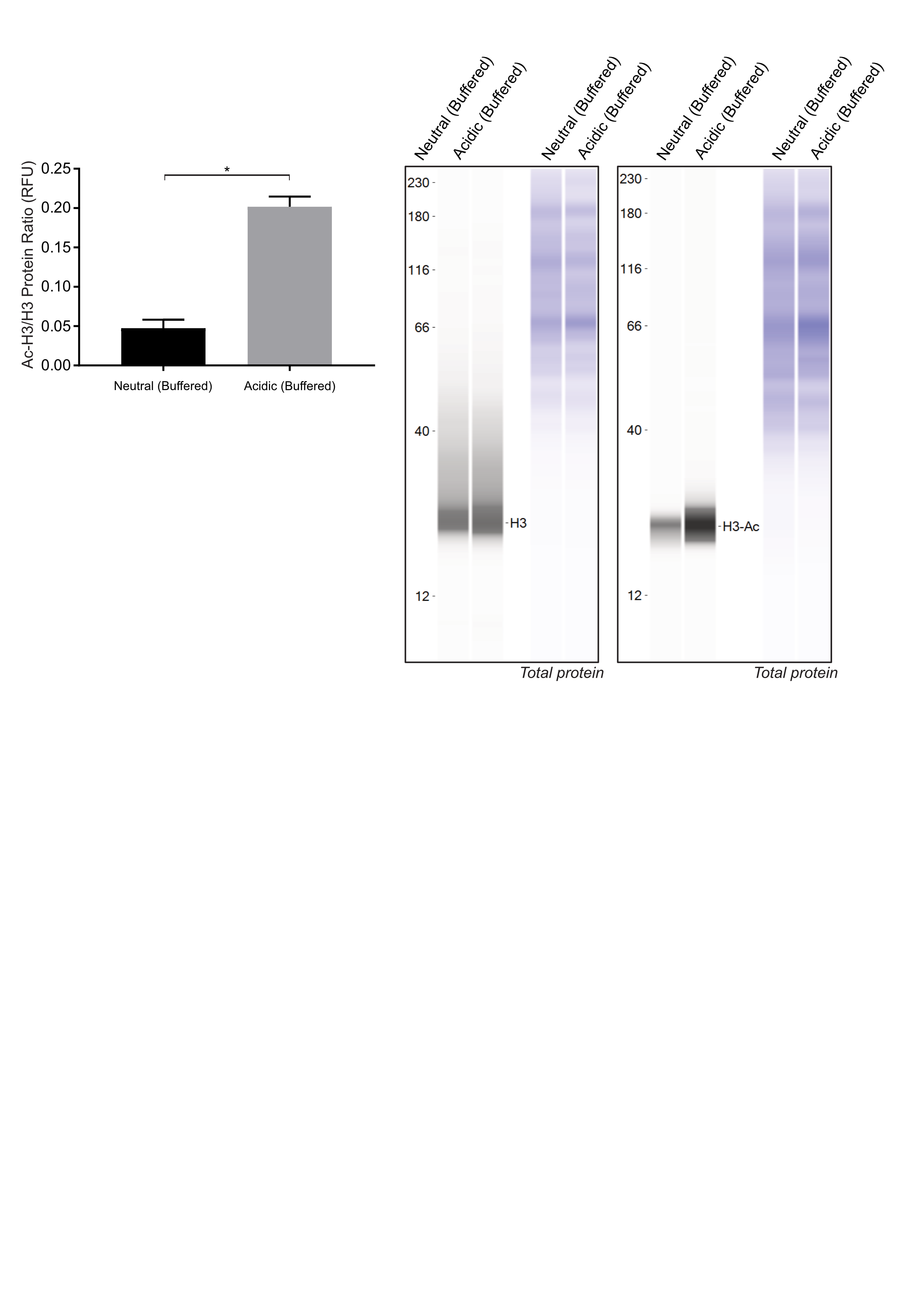


## **Suppl. Fig. 3.**

## Acidosis increases acetylation in acidic (buffered, pH 6.8). Left panel, acetylated/non-acetylated H3 ratio quantification (*p<0.05, n=6) of H3 and H3-Ac in 143B spheroids under neutral vs acidic conditions (buffered pH 6.8); right panel, representative images. One-tailed Mann-Whitney test, mean±SEM.

##


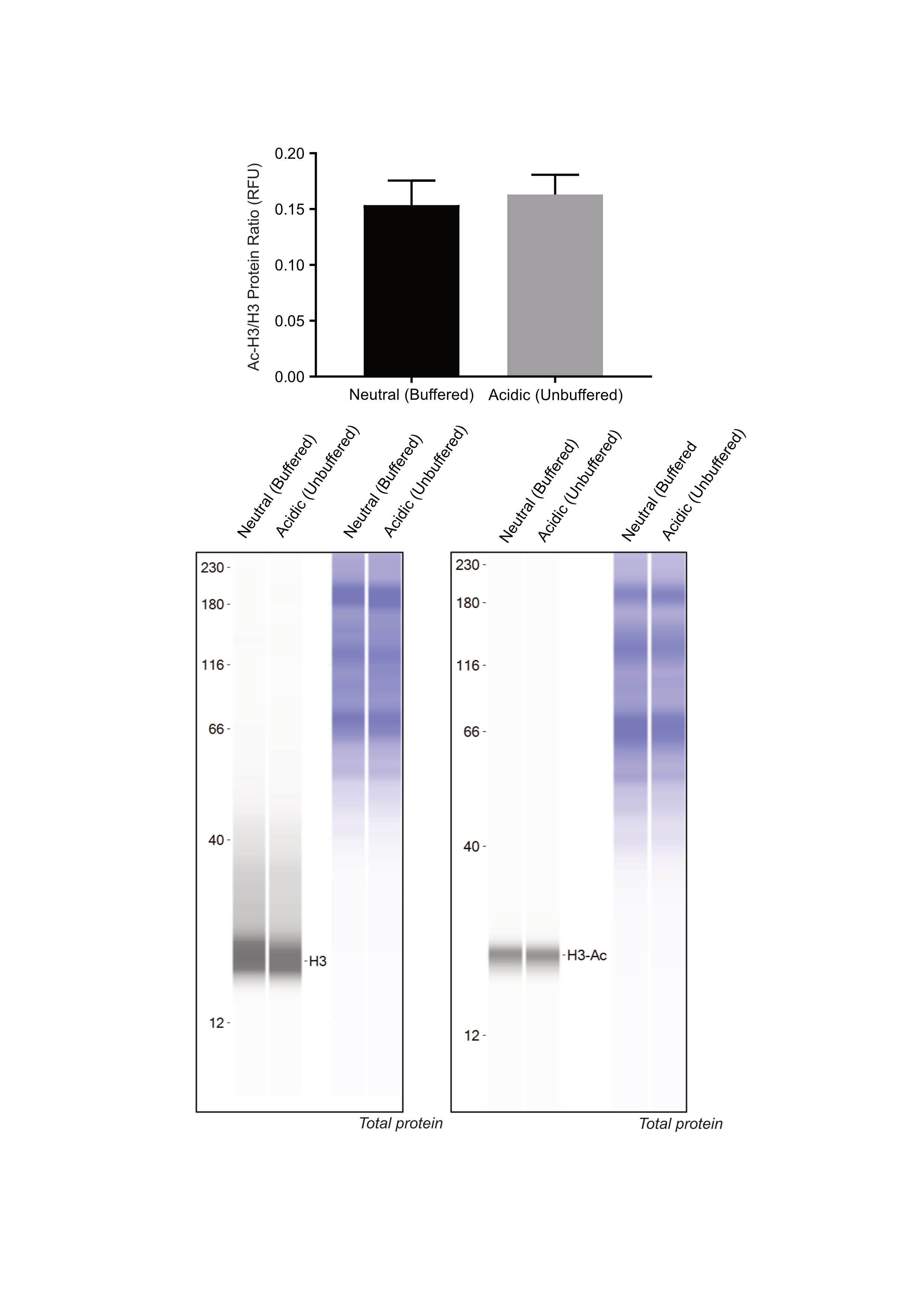


## **Suppl. Fig. 4.**

## Acetylation of H3 does not change in MG-63 spheroids. Upper: acetylated/non-acetylated H3 ratio quantification after 72 hrs of exposure to acidic (unbuffered) conditions of MG-63, as revealed by representative capillary western blot. Mean±SEM.


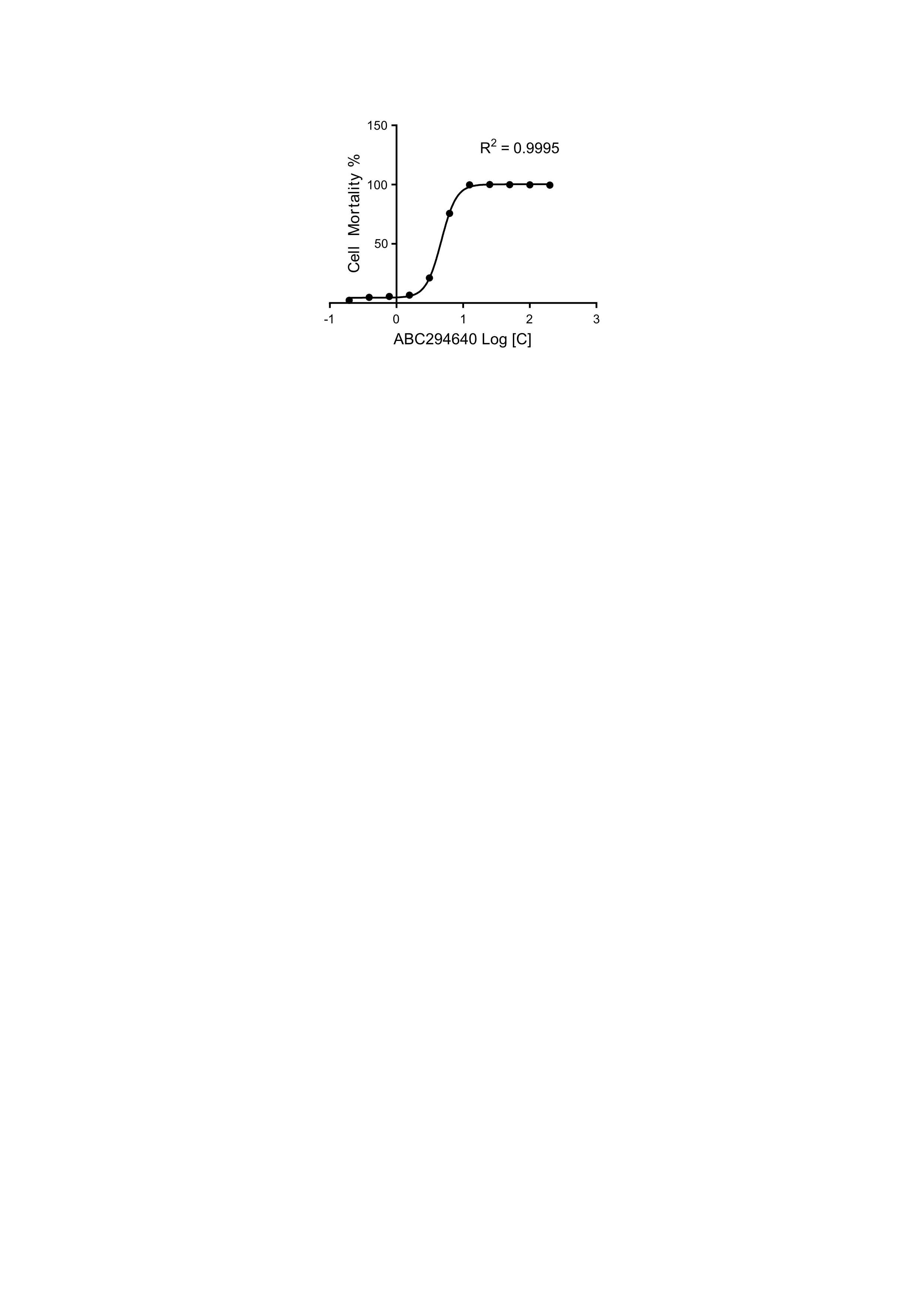


## **Suppl. Fig. 5.**

IC50 for ABC294640, assessed as 5,6 μM. Nonlinear fit of cell mortality rate over logarithm of treatment concentration.


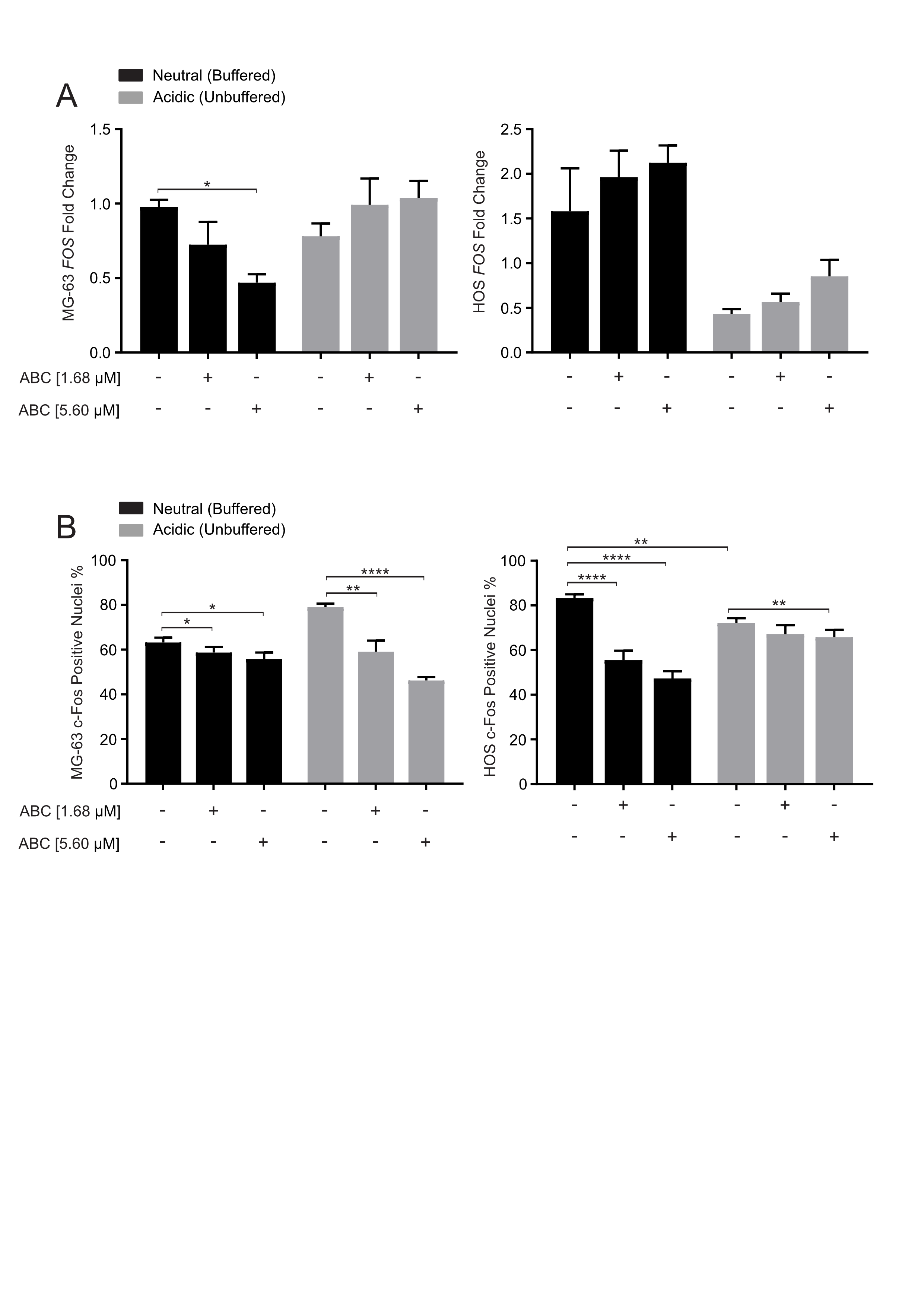


## **Suppl. Fig. 6.**

Effect of ABC294640 and DMS treatment on *FOS* expression as revealed by Real-Time PCR (A), and FOS nuclear localization as revealed by immunofluorescence (B) in MG-63 and HOS cell lines. Mean±SEM, (*p<0.05; **p<0.01, ***p<0.001; ****p<0.0001 n=8).


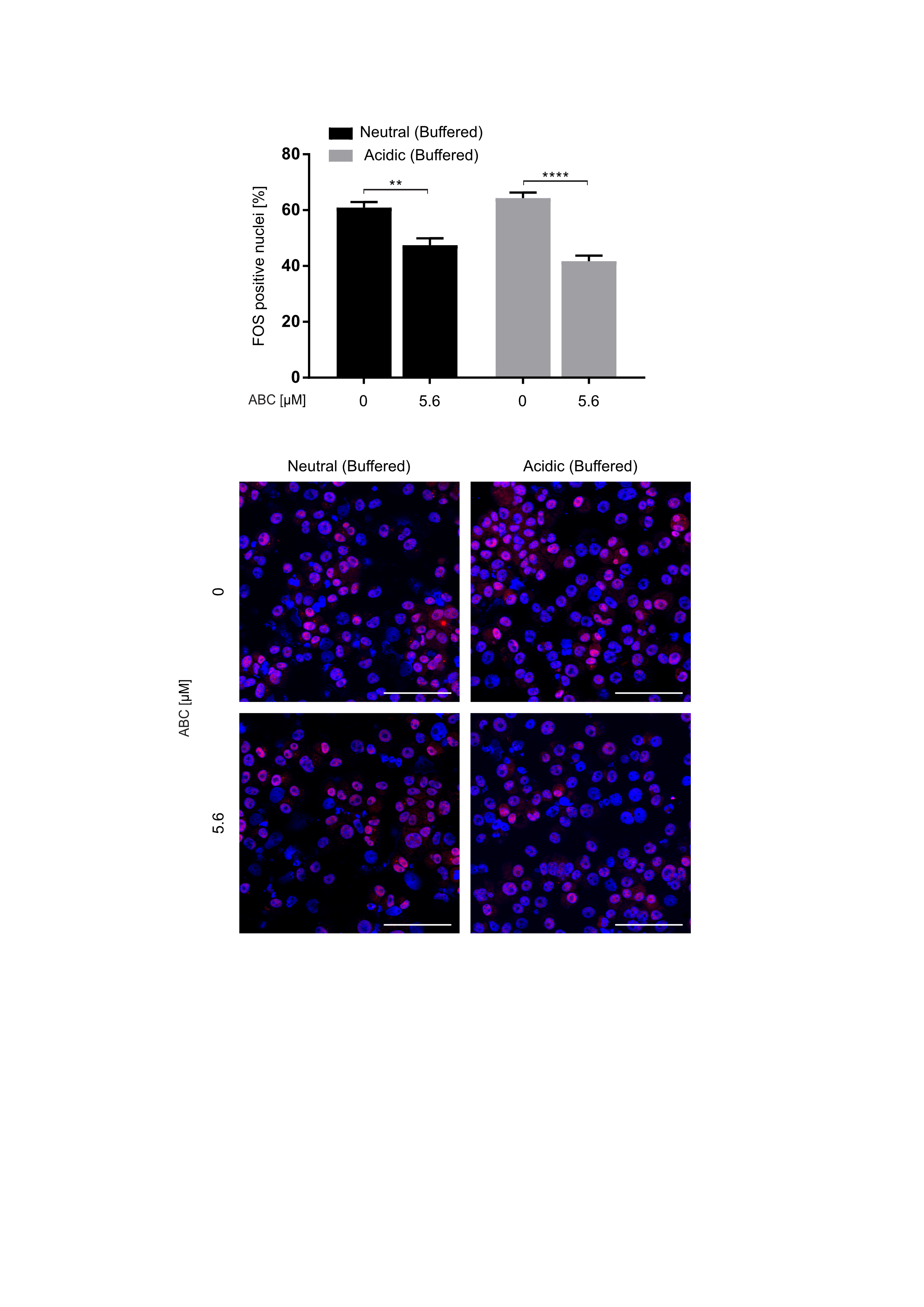


## **Suppl. Fig. 7.**

ABC294640 reduces FOS expression and nuclear localization in acidic (buffered, pH 6.8) conditions. Top: representative FOS immunofluorescence in 143B spheroids in neutral vs unbuffered medium over time by immuofluorescence (red, nuclei were counterstained with bisBenzimide H33258) (scale bar 100 µm); Right: FOS positive nuclei (%) (**p<0.01, ****p<0.0001 n=5)
